# Demonstrating the Psychological Aspects of Stressors and Abusive Supervision Behavior: Attainment of Sustainability Under the Rubric of Resources Theory

**DOI:** 10.3389/fpsyg.2020.00293

**Published:** 2020-03-13

**Authors:** Zeying Li, Bin He, Xu Sun, Yun Zhang

**Affiliations:** ^1^School of Management, Guangdong University of Technology, Guangzhou, China; ^2^School of Computer Information Engineering, Hanshan Normal University, Chaozhou, China; ^3^School of Economics and Management, Wuzhou University, Wuzhou, China

**Keywords:** challenge stressors, hindrance stressors, emotional intelligence, abusive supervision behavior, conservation of resources theory

## Abstract

This article builds on the conservation of resources (COR) theory and the challenge–hindrance stressors framework to propose a model for understanding and investigating why and when these two distinct categorized stressors similarly promote the display of abusive supervision behavior. The data from 228 supervisors and subordinates are selected by using the time-lagged method. Prior to hypothesis testing, we first conducted confirmatory factor analyses (CFAs) of the proposed models in Lisrel software. Hierarchical regression analysis revealed why and when two distinct categorized stressors positively relate to ego depletion and thus, in turn, promote the display of abusive supervision behavior. The bootstrap methods confirmed the mediating effect of ego depletion and the moderated-mediation role of emotional intelligence (EI). The findings show that both challenge and hindrance stressors are positively related to ego depletion and that ego depletion is positively associated with abusive supervision behavior. Results suggest that challenge and hindrance stressors have similar positive effects on abusive supervision behavior via the mediating effect of ego depletion. In addition, we find that supervisors’ EI weakens the positive relationship between challenge stressors and ego depletion, and it also weakens the positive relationship between hindrance stressors and ego depletion. This study extends the current literature by directly testing resource depletion as a mediating mechanism and resource replenishment as a boundary condition of the effect of work stressors.

## Introduction

Abusive supervision behavior has drawn much attention from several researchers in the last few decades (Mitchell et al., 2015; Camps et al., 2018; Qin et al., 2018). It refers to subordinates’ perception of the extent to which supervisors engage in the sustained display of hostile verbal and non-verbal behaviors, excluding physical contact (Tepper, 2000). Empirical evidence reveals that abusive supervision behavior has a host of deleterious effects on employees (Han et al., 2017) and organizations (Mitchell and Ambrose, 2007; Frieder et al., 2015; Mackey et al., 2017). Given the hindrance role of this behavior in the sustainable development of organizations, an increasing number of scholars have sought to probe its antecedents in order to lessen its deleterious effects. Prior empirical evidence has identified that some work stressors (e.g., exceedingly difficult job goals, task difficulty, role overload) are the antecedents of abusive supervision behavior (Burton et al., 2012; Mawritz et al., 2014; Eissa and Lester, 2017). These studies agree that work stressors often positively relate to negative behavior. More specifically, work stressors first evoke negative emotions, such as anger, anxiety, or frustration, which in turn predict abusive supervision behavior. Among the work on this stream of studies, scholars focus more on the effect of single-dimensional work stressors, that is how single-dimensional work stressors influence emotional response, thus further influencing abusive supervision behavior. Although these studies reveal that work stressors can predict abusive supervision behavior, they do not distinguish the different categories of work stressors. Hence, those findings can’t well address the question of why and when distinct categorized stressors lead to abusive supervision behavior in a similar way.

Having noticed that some stressors have positive outcomes, while some others have negative outcomes, Cavanaugh et al. (2000) decided to split stressors into two distinct categories: challenge stressors and hindrance stressors, which would result in positive outcomes or negative outcomes, respectively. Workload, time urgency, job responsibility, and job complexity were considered challenge stressors, whereas red tape, role ambiguity, role conflict, and hassles were labeled as hindrance stressors (Rodell and Judge, 2009). Given how meaningful and useful these categorizations are in the workplace, stress researchers have devoted great effort to probe their outcomes, so as to effectively cope with challenge and hindrance stressors in the workplace (O’Brien and Beehr, 2019). From different perspectives, such as a psychology lens (Rodell and Judge, 2009), attitude lens (LePine et al., 2005; Wallace et al., 2009; Yao et al., 2015), and behavior lens (Rodell and Judge, 2009), previous studies investigated the possible outcomes of challenge and hindrance stressors. Following the challenge–hindrance stressors framework, these studies have found that these two distinct categorized stressors have different effects on resilience, emotional reaction, job satisfaction, job performance, cynicism, and inefficacy (LePine et al., 2005; Wallace et al., 2009; Yao et al., 2015; Crane and Searle, 2016), while having similar effects on emotional exhaustion and depression (LePine et al., 2005; Podsakoff et al., 2007; Yao et al., 2015). In a word, these studies focus more on different effects on some behaviors or attitudes and the similar effect of challenge–hindrance stressors on some psychology variables (i.e., psychology strain), ignoring that different types of stressors may have a similar effect on the same behavior through similar psychological process. Therefore, the current study pays more attention to the relationship between challenge–hindrance stressors and abusive supervision behavior, because these two categories may affect abusive supervision behavior in a similar way through similar psychological processes. Consequently, in the current study, we take the first step to explore why these two distinct categorized stressors have a similar psychological process in abusive supervision behavior. Second, we investigate when these two distinct categorized stressors would generate a similar psychological process, thus further predicting abusive supervision behavior.

Conservation of resources (COR) theory has long been an important theoretical foundation for understanding the mechanism of work stressors in behavior (Hobfoll, 1989), which declares that resource loss is ordinary in our life, and people must then invest resources in order to protect against resource loss, recover from losses, and gain resources. Under the condition of challenge stressors, individuals will invest psychological resources, such as effort, attention, and willpower, to meet the requirements of work and achieve personal growth. And under the condition of hindrance stressors, individuals will also invest psychological resources to overcome hindrance stressors, for they are detrimental to job performance goals and career growth. Such psychological resources can be also called self-regulatory resources when they are used to self-regulate or self-control (Baumeister et al., 1998). Self-regulation refers to the mental abilities possessed by individuals to control and regulate their own emotions, behaviors, and psychological states. According to COR theory, individuals have a limited self-regulatory resource reserve that can be mobilized (Shapiro et al., 1997; Hobfoll, 2002). As discussed above, dealing with challenge stressors and hindrance stressors is a typical process of self-regulation, which will consume the finite pool of self-regulatory resources. Self-regulation is the key to effectively regulating behavior because people have a finite pool of psychological resources to fuel positive behaviors and block out negative behaviors; thus, it often results in resource loss. On one hand, resource loss will result in reduced capacity for further self-regulation (Baumeister et al.,1998); on the other hand, people will also invest fewer resources to self-regulate in order to protect against further resource loss (Hobfoll, 1989). In a word, resource loss that comes from dealing with challenge stressors and hindrance stressors will cause individuals to experience self-regulation impairment, a state of self-regulatory resource depletion (Tepper et al., 2017), thereby increasing the likelihood of a supervisor’s abusive behavior.

Therefore, COR provides the theoretical basis for understanding why and when challenge–hindrance stressors have a similar effect on abusive supervision behavior. The previous study has proposed that work stressors (i.e., role overload) are positively associated with abusive supervision through psychological resource depletion (Eissa and Lester, 2017). However, to date, there has been no empirical research on the mediating mechanism of specific resource depletion variables between work stressors and abusive supervision behavior. The strength model of self-control provides a specific resource depletion variable, called ego depletion, which can well represent a depletion state of self-regulatory resources (Baumeister et al., 2007). Therefore, we propose that two distinct categorized stressors may positively relate to abusive supervision behavior via ego depletion. More specifically, individuals invest their limited self-regulatory resources to cope with work stressors, which further leads to ego depletion (Baumeister et al., 1998; Baumeister et al., 2007; Hagger et al., 2010). Individuals in this state have difficulty investing resources to self-regulate and thus are more likely to exhibit abusive behavior (Barnes et al., 2015; Lin et al., 2016). One purpose of our study is to examine the mediating role of the specific resource depletion variable between work stressors and abusive supervision behavior and to further verify the effectiveness of the explanation of the resource depletion mechanism. Resource depletion of Hierarchical CEO interferes with successor selection and innovation decision (Sarfraz et al., 2019b).

However, one should not assume that all individuals respond to the same work stressors in the same way. Previous studies suggest that task valence (a perception of task value), exercise (a leisure activity) (Burton et al., 2012), and supervisor personality (Eissa and Lester, 2017) help explain why individuals’ reactions to work stressors may vary. Such previous studies have neglected the possible moderating role of variables that reflect differences in self-regulatory resources. From the perspective of COR theory, emotional intelligence (EI), which is defined as “the ability to carry out accurate reasoning about emotions and the ability to use emotions and emotional knowledge to enhance thought” (Salovey and Mayer, 1990), can be viewed as an individual resource characteristic variable (Koubova and Buchko, 2013) that can reflect individual differences in self-regulatory resources. This is because EI, first, as an individual’s mental ability to cognize and evaluate the emotional states of oneself and others, as well as to use and express emotions (Wong and Law, 2002), is the key resource to help individuals to self-regulate in stressful situations. That is, EI is a kind of self-regulatory resource. Second, EI can help individuals gain more role resources from playing roles successfully, as well as gain more contextual resources from interpersonal interaction. Therefore, EI can well reflect individual differences in self-regulatory resources. A high level of EI can effectively replenish the depletion of an individual’s self-regulatory resources; thus, it can well explain and confirm the role of the resource replenishment mechanism in coping stressors. That is, a high level of EI can help individuals weaken the depletion of self-regulatory resources under stressful conditions. Specifically, we propose that the relationship between challenge–hindrance stressors and ego depletion is weaker when supervisors have a higher level of EI. Accordingly, the present study attempts to expand the application of EI by studying its weakening effect on stressors and ego depletion, thus confirming that self-regulatory resources can help individuals deal with challenge–hindrance stressors more effectively, and the existence and effectiveness of the resource replenishment mechanism.

Our study provides several primary theoretical contributions to the literature. First, this study contributes to the enrichment of research on the relationship between challenge–hindrance stressors and abusive supervision. Furthermore, previous stress studies paid more attention to different effects on some behaviors or attitudes, and the similar effect of challenge–hindrance stressors on some psychology variables, ignoring that different categories of stressors may have a similar effect on the same behavior through a similar psychological process. Thus, we examine why and when challenge–hindrance stressors have similar effects on abusive supervision. Our findings not only can offer useful insights to understand the complex relationship between challenge–hindrance stressors and abusive supervision but also confirm that positive stressors (i.e., challenge stressors) may also result in negative leadership behavior (i.e., abusive supervision). Second, we contribute to empirical research verifying the explanatory power of the resource depletion mechanism by focusing on a specific resource depletion variable: ego depletion. Although a previous study has noted the role of resource depletion between work stressors and abusive supervision behavior (Eissa and Lester, 2017), there is no empirical research on the mediating mechanism of specific resource depletion variables. In the current study, we identify that ego depletion can serve as a specific resource depletion variable, which comes from the strength model of self-control (Baumeister et al., 2007). Furthermore, we confirm the mediating role of ego depletion, thus further revealing why challenge–hindrance stressors have a similar effect on abusive supervision behavior. Our research not only shows that resource depletion may be a key mechanism for the similar effects of challenge–hindrance stressors but also offers empirical evidence that ego depletion can be viewed as a specific resource depletion variable. Third, we enrich the existing research on EI by considering the resource replenishment mechanism as the boundary condition of the indirect relationship between work stressors and abusive supervision behavior. As stated earlier, prior studies have not focused on the possible moderating role of variables that reflect individual differences in self-regulatory resources. EI, first, itself is a kind of self-regulatory resource and, second, can help individuals gain more resources from playing roles successfully and interpersonal interaction. That is, EI can well reflect individual differences in self-regulatory resources. Thus, we recognize the necessity to examine the possible moderating role of supervisors’ EI. Our findings suggest that high EI can effectively replenish the depletion of an individual’s self-regulatory resources, compared to low EI. Therefore, our study expands the research on the boundary conditions of abusive supervision behavior by unveiling a new type of moderating mechanism of resource replenishment from the resource’s lens. Our findings not only confirm that self-regulatory resources can help individuals deal with challenge–hindrance stressors more effectively but also confirm the existence and effectiveness of the resource replenishment mechanism. Finally, we extend the research adopting the resource lens by integrating both mediating and moderating mechanisms into a single model, providing an account of how challenge–hindrance stressors affect abusive supervision behavior in a similar way and for whom work stressors are most damaging, as well as developing practical implications by identifying ways that can be used to mitigate the effects of work stressors.

## Theoretical Framework and Hypothesis Development

### The Relationship Between Challenge–Hindrance Stressors and Abusive Supervision Behavior

COR theory holds that resource loss is ordinary in our lives, and then people must invest resources in order to protect against resource loss, recover from losses, and gain resources (Hobfoll, 1989). The resource perspective can be applied to explain how supervisors respond to challenge–hindrance stressors, emphasizing the resource depletion process of dealing with the stressor. Based on the COR theory, individuals have a limited self-regulatory resource reserve that can be mobilized (Shapiro et al., 1997; Hobfoll, 2002). According to prior research, activities such as controlling unwanted behavior, managing emotions (Muraven and Baumeister, 2000), and coping with stress are required for self-regulation (Baumeister et al., 2007), and such processes often consume individuals’ limited self-regulation resources (Muraven and Baumeister, 2000). Furthermore, as self-regulatory resources are continuously decreasing over time, people will become stuck in resource depletion or lose the ability to effectively regulate their behaviors (Beal et al., 2005; Wang et al., 2013). The following are two typical examples of work stressors: Challenge stressors are viewed by an individual as surmountable work-related demands that are prone to assist with achievements at work and are likely to be associated with personal potential gains and growth (Cavanaugh et al., 2000). Examples of challenge stressors include workload, time urgency, job responsibility, and job complexity (Rodell and Judge, 2009). In contrast, hindrance stressors are viewed by an individual as insurmountable work-related demands that interfere with achievements at work and are often viewed as constraints or obstacles to personal potential gains, growth, or achievements (Cavanaugh et al., 2000). Examples of hindrance stressors include red tape, role ambiguity, role conflict, and hassles (Rodell and Judge, 2009). Workplace ostracism is positively associated with stress (Sarfraz et al., 2019a).

Ego depletion refers to a depletion state of the limited pool of self-regulatory resources that individuals use to perform regulatory abilities (Baumeister et al., 1998; Baumeister et al., 2007; Hagger et al., 2010). Dealing with challenge and hindrance stressors will consume the limited pool of self-regulatory resources, leaving actors feeling resource depletion and a lack of resources to self-regulate, which is referred as a state of “ego depletion” (Baumeister et al., 1998; Baumeister et al., 2007; Hagger et al., 2010). Specifically, in order to obtain the development and growth brought by challenge stressors, individuals need to invest sustained attention, willpower, and effort to complete difficult tasks, to struggle with time urgency, to assume responsibilities, or to settle complex problems. Such activities deplete individuals’ self-regulatory resources and further lead to a state of ego depletion (Baumeister et al., 2007; Loseman and van den Bos, 2012; Singh and Göritz, 2018). Similarly, when facing hindrance stressors, individuals need to invest sustained attention, willpower, and effort to cope with red tape, to solve problems of role ambiguity and role conflict, or to control the negative emotions evoked by these stressors. Such activities also deplete individuals’ self-regulatory resources and further lead to a state of ego depletion (Baumeister et al., 2007; Loseman and van den Bos, 2012; Singh and Göritz, 2018). As such, based on the COR theory, it seems plausible that both challenge and hindrance stressors are positively related to ego depletion.

*Hypothesis 1a:* Dealing with challenge stressors is positively related to ego depletion.
*Hypothesis 1b*: Dealing with hindrance stressors is positively related to ego depletion.

As a typical negative leadership behavior, abusive supervision behavior is related to a broad range of deleterious effects on a host of employee and organizational outcomes (Yam et al., 2016; Mackey et al., 2017). Because of its harmfulness, supervisors often invest their limited pool of self-regulatory resources to avoid engaging in such behavior. When supervisors have abundant self-regulatory resources, they tend to perform well in self-regulatory activities. But when they face self-regulatory resource depletion, they tend to perform poorly in self-regulatory activities. In other words, when individuals have abundant self-regulatory resources, they may avoid engaging in abusive behavior through self-regulation. But when the individual’s self-regulatory resources are depleted, they are more likely to engage in abusive behavior. This is because, first, they do not have enough resources to regulate and control negative behaviors (Muraven and Baumeister, 2000; Baumeister et al., 2007). Second, they will also invest fewer resources to self-regulate in order to protect against further resource loss (Hobfoll, 1989). Given that ego depletion represents a state of self-regulatory resource depletion, it seems plausible believe that supervisors more easily engage in abusive behavior when they experience more ego depletion. Specifically, when supervisors experience more ego depletion, then they may have insufficient resources or invest fewer resources to self-regulate in order to curtail abusive supervision behavior during subsequent interactions with their subordinates (Barnes et al., 2015; Lin et al., 2016; Yam et al., 2016; Welsh et al., 2018). We propose the following:

*Hypothesis 2*: Ego depletion is positively related to abusive supervision behavior.

From the perspective of resource depletion, challenge and hindrance stressors may similarly precipitate abusive supervision behavior via the mediating effect of ego depletion. Specifically, when facing challenge stressors, individuals invest their limited pool of self-regulation resources to acquire potential development and growth; and when facing hindrance stressors, individuals should, even more, invest their limited pool of self-regulatory resources to control their negative emotions or overcome obstructive feelings. Thus, supervisors are less likely to exercise self-control to counteract abusive tendencies because work stressors result in constant consumption of limited self-regulatory resources (Liu et al., 2015; Lam et al., 2017). As such, it seems plausible that both challenge and hindrance stressors result in ego depletion, which in turn increases the likelihood of abusive supervision behavior. Organizational justice partially mediates between an employee’s perception of corporate social responsibility and employee outcomes (Sarfraz et al., 2018).

*Hypothesis 3a*: Challenge stressors have a positive, indirect effect on abusive supervision behavior via ego depletion of the supervisor.
*Hypothesis 3b*: Hindrance stressors have a positive, indirect effect on abusive supervision behavior via ego depletion of the supervisor.

### Moderating Role of EI

Conservation of resources theory also can be applied to explain why individuals’ reactions to workplace stressors may vary. When an individual has a larger psychological resource pool, he or she may have more self-regulatory resources to cope with stressors, thus weakening the positive effect of work stressors on ego depletion. Therefore, self-regulatory resources can be the boundary conditions of two different types of work stressors and abusive supervision behavior. Then, which variable can reflect the individual differences in self-regulatory resources?

Mayer et al. (2008) defined EI as the ability to carry out accurate reasoning emotions and use these emotions and emotional knowledge to enhance thoughts. In short, EI is a series of interpersonal and intrapersonal mental abilities that helps people to understand their own and others’ emotions (Austin et al., 2007), thus further regulating their own feelings, behavior, and actions by processing their emotions. According to previous research, EI, which is an individual resource characteristic variable, can be regarded as a key psychological resource to help individuals to self-regulate in stressful situations. That is, EI itself is a kind of self-regulatory resource. To a certain extent, it can replenish the continuous depletion of an individual’s self-regulatory resources when he or she performs self-regulatory activities, such as coping with work stressors. On the other hand, EI can help individuals gain more resources that they use to replenish the depletion of their self-regulatory resources. More specifically, individuals with high EI can gain more role resources by successfully playing various roles, experience more positive emotions, and gain more contextual resources from interpersonal interaction. In a word, EI can well reflect individual differences in self-regulatory resources. Therefore, individuals with high EI can effectively replenish the depletion of their self-regulatory resources. As a result, although they consume self-regulatory resources when dealing with challenge stressors and hindrance stressors, they still have sufficient resources to self-regulate, which, in turn, weakens the positive effect of work stressors on ego depletion. In contrast, individuals with low EI cannot effectively replenish the depletion of their self-regulatory resources. As a result, they have insufficient resources to self-regulate, which, in turn, strengthens the positive effect of work stressors on ego depletion.

In line with this reasoning, we propose the following:

*Hypothesis 4a*: EI moderates the relationship between challenge stressors and ego depletion such that the relationship is weaker for supervisors with high EI than for those with low EI.
*Hypothesis 4b*: EI moderates the relationship between hindrance stressors and ego depletion such that the relationship is weaker for supervisors with high EI than for those with low EI.

Finally, as we argued for the indirect effect of two different types of stressors (i.e., challenge stressors and hindrance stressors) on abusive supervision behavior through supervisors’ ego depletion as well as for the moderating role of supervisors’ EI, we propose a first-stage moderated-mediation model in order to illustrate the combined role of the above-discussed constructs in the display of abusive supervision behavior. That is, we expect that the weakness of this indirect effect will vary among different levels of supervisors’ EI. More specifically, supervisors’ EI negatively moderates the relationship between challenge stressors (or hindrance stressors) and abusive supervision behavior via ego depletion. When supervisors have high EI, the indirect effect between challenge stressors (or hindrance stressors) and abusive supervision behavior via ego depletion is weaker. When supervisors have low EI, the indirect effect between challenge stressors (or hindrance stressors) and abusive supervision behavior via ego depletion is stronger.

*Hypothesis 5a*: The indirect effect of challenge stressors on abusive supervision behavior via ego depletion will be weaker when supervisors have high EI rather than low EI.
*Hypothesis 5b*: The indirect effect of hindrance stressors on abusive supervision behavior via ego depletion will be weaker when supervisors have high EI rather than low EI.

**FIGURE 1:**
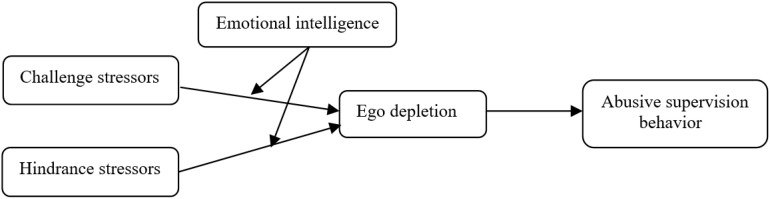
The conceptual model.

In conclusion, the conceptual model of this study is shown in Figure 1.

## Materials and Methods

To obtain multi-regional and multi-industry survey data, we asked our team members to help recruit participants among their friends who play a supervisory role in their company. When their friends who had at least one subordinate responded to our request, we invited them and their direct subordinates to participate in our study. To allow matching, all participants were coded with the help of our team members, for example, a code consisting of “LD” and a serial number (i.e., LD001) for a supervisor and a code consisting of “EP” and the same serial number (i.e., EP001) for his or her direct subordinate. Questionnaires were distributed to respondents by e-mail containing a unique link code for them to the online questionnaire. To ensure data quality, participants were informed that the survey would be conducted confidentially and anonymously, and written informed consent was inferred through the completion of the survey. Participants were compensated for completing the survey.

To reduce common method bias (CMB) (Podsakoff et al., 2012) and to avoid the retrospective bias of the long interval, we conducted a multi-wave survey with a gap of 2 or 3 days according to Lin et al. (2016). At time 1 (Sunday morning), supervisors were asked to report their EI and demographic information. At time 2 (Tuesday afternoon), the challenge–hindrance stressors and ego depletion of supervisors for the past 2 days were assessed. Finally, the perception of abusive supervision behavior over the past 3 days and the demographic information of subordinates were assessed at time 3 (Friday afternoon).

In total, 348 supervisors agreed to participate in our study. We received 313 valid supervisors’ questionnaires at T1, and the response rate was 89.94%. At T2, we distributed 313 questionnaires to those who replied effectively at T1 and received 283 valid supervisors’ questionnaires; the response rate was 90.42%. At T3, we asked 1 of the 283 valid supervisors’ subordinates to complete the subordinate questionnaire, and we received 228 valid subordinates’ questionnaires; the response rate was 80.57%. Finally, we matched 228 dyads data from the supervisor and subordinate one by one. Of the supervisor sample, 55.26% were male, with an average age of 36.61 years (SD = 6.12) and an average education of 1.05 (0 = college degree or below, 1 = university degree, 2 = master’s degree or above, SD = 0.42). Of the subordinate sample, 42.54% were male, with an average age of 30.57 years (SD = 6.33), an average education of 0.75 (SD = 0.43), and an average tenure with their supervisor of 2.50 years (SD = 1.82).

### Measures

The used measures were identical to those that have been widely used in previous studies (detailed in the Supplementary Appendix). Most items were rated on a five-point scale.

#### Challenge–Hindrance Stressors

We used the 11-item measure developed by Cavanaugh et al. (2000) to test challenge stressors (items 1–6, α = 0.88) and hindrance stressors (items 7–11, α = 0.86). Participants were asked to indicate the extent to which the statements produced stress at work during the past 2 days and rated items on a scale ranging from 1 (*no stress*) to 5 (*a great deal of stress*). A sample item for challenge stressors was “The amount of time I spend at work,” and a sample item for hindrance stressors was “The amount of red rape I need to go through to get my job done.”

#### Ego Depletion

Ego depletion was measured with a five-item short scale that was used and validated by Johnson et al. (2014). These items originally came from the work of Twenge et al. (2004) and published by Christian and Ellis (2011). Participants reported the extent to which each statement represented how they felt during the past 2 days and rated items (α = 0.89) on a scale ranging from 1 *(very slightly or not at all)* to 5 (*very much)*. A sample item was “My mental energy is running low.”

#### Emotional Intelligence

We used a 16-item version of the EI scale of Wong to measure EI (α = 0.93), including four dimensions: Self-emotion appraisal (SEA), Others’ emotion appraisal (OEA), Use of emotion (UOE), Regulation of emotion (ROE) (Wong and Law, 2002). Participants reported to what extent they agreed with each statement on a scale ranging from 1 (*strongly disagree*) to 5 (*strongly agree)*. A sample item was “I really understand what I feel.”

#### Abusive Supervision Behavior

In our study, subordinates rated their direct supervisor on abusive supervision behavior during the past 3 days by using the five-item short scale (α = 0.81) of Mitchell and Ambrose (2007). Sample items included “My supervisor ridicules me” and “My supervisor tells me I’m incompetent” on a five-point scale, from 1 (*never*) to 5 (*very often*).

#### Control Variables

The results from prior studies suggest that supervisors’ gender (0 = female, 1 = male), age, and education (0 = college degree or below, 1 = university degree, 2 = master’s degree or above), as well as subordinates’ tenure with the supervisor, are related to abusive supervision behavior (Breevaart and de Vries, 2017). In order to test the model rigorously, we also included supervisors’ gender, age, and education, as well as subordinates’ tenure with the supervisor, as control variables.

### Measurement Model

Prior to hypothesis testing, we first conducted confirmatory factor analyses (CFAs) of the proposed models in Lisrel software, and the results are presented in Table 1. According to Little et al. (2002), when the number of construct items is relatively large while sample sizes are relatively small, parcels of items can be used to simplify the measurement model. And one technique for building parcels is to use the dimensions as the grouping criteria to create parcels. Therefore, we used four dimensions of EI as four parcels because our sample sizes were relatively small. As shown in Table 1, the proposed five-factor model (i.e., challenge stressors, hindrance stressors, ego depletion, EI, and abusive supervision behavior) revealed an acceptable fit (Model 1): [χ2/df = 1.35, comparative fit index (CFI) = 0.98, incremental fit index (IFI) = 0.98, root mean square error of approximation (RMSEA) = 0.04] and fit better than alternative models (e.g., Models 2 to 10). The test showed that the discriminant validity of our focal variables was significant.

**TABLE 1:** Comparison of measurement models.

Model	Descriptions	χ^2^	*df*	χ*^2^/df*	Δχ^2^	CFI	IFI	RMSEA
Model 1	Five factors: CS, HS, ED, EI, AS	358.97	265.00	1.35		0.98	0.98	0.04
Model 2	Four factors: CS, HS, ED + EI, AS	714.25	269.00	2.66	355.28**	0.94	0.94	0.09
Model 3	Four factors: CS, HS, ED, EI + AS	766.16	269.00	2.85	407.19**	0.94	0.94	0.09
Model 4	Four factors: CS, HS + ED, EI, AS	836.20	269.00	3.11	477.23**	0.94	0.94	0.10
Model 5	Four factors: CS + HS, ED, EI, AS	1,096.35	269.00	4.08	737.38**	0.91	0.91	0.12
Model 6	Three factors: CS, HS + ED, EI + AS	1,233.63	272.00	4.54	874.66**	0.89	0.89	0.13
Model 7	Three factors: CS + HS, ED + EI, AS	1,455.05	272.00	5.35	1,096.08**	0.87	0.87	0.14
Model 8	Two factors: CS + HS, ED + EI + AS	1,783.80	274.00	6.51	1,424.83**	0.83	0.83	0.16
Model 9	Two factors: CS + HS + ED, EI + AS	2,163.27	274.00	7.90	1,804.30**	0.81	0.81	0.17
Model 10	One factor: CS + HS + ED + EI + AS	2,305.27	275.00	8.38	1,946.30**	0.78	0.78	0.18

N = 228. CS = challenge stressors, HS = hindrance stressors, ED = ego depletion, EI = emotional intelligence, AS = abusive supervision behavior, RMSEA = root mean square error of approximation, CFI = comparative fit index, IFI = incremental fit index. **p < 0.01.

## Study Results

### Correlations and Descriptive Statistics

Table 2 presents the descriptive statistics and correlations among the study variables. The results indicated that challenge stressors (*r* = 0.27, *p* < 0.01) and hindrance stressors (*r* = 0.40, *p* < 0.01) are positively correlated with abusive supervision behavior. Challenge stressors (*r* = 0.28, *p* < 0.01) and hindrance stressors (*r* = 0.48, *p* < 0.01) are positively correlated with ego depletion, and ego depletion is positively correlated with abusive supervision behavior (*r* = 0.44, *p* < 0.01).

**TABLE 2:** Descriptive statistics and correlations.

Variable	*M*	*SD*	1	2	3	4	5	6	7	8
CS	3.65	0.76	(0.88)							
HS	3.00	0.84	0.30**	(0.86)						
ED	3.05	0.76	0.28**	0.48**	(0.89)					
EI	3.68	0.51	−0.15*	−0.31**	−0.35**	(0.93)				
AS	2.61	0.54	0.27**	0.40**	0.44**	−0.33**	(0.81)			
Gender^a^	0.55	0.50	–0.03	–0.08	–0.06	0.09	–0.12			
Age	36.61	6.12	–0.04	–0.05	–0.05	0.03	–0.01	0.21**		
Education^b^	1.05	0.42	–0.05	–0.01	0.01	0.19**	–0.05	0.05	−0.15*	
Tenure^c^	2.50	1.82	–0.03	0.02	–0.01	0.12	0.02	0.06	0.23**	0.02

N = 228. Coefficient alphas are given in parentheses on the diagonal. Tenure = subordinate’s tenure with the supervisor. ^a^0 = female, 1 = male. ^b^0 = college degree or below, 1 = university degree, 2 = master’s degree or above. ^c^This was completed only by subordinates and is in yearly units. *p < 0.05, **p < 0.01.

### Hypothesis Testing

Hierarchical regression analysis was adopted to test Hypotheses 1–4 (including a and b). First, as presented in Table 3, Hypothesis 1a, which predicates a positive relationship between challenge stressors and ego depletion, is supported (β = 0.15, *p* < 0.05, M1), and Hypothesis 1b, which predicates a positive relationship between hindrance stressors and ego depletion, is also supported (β = 0.39, *p* < 0.001, M1). Second, Hypothesis 2, which predicates a positive relationship between ego depletion and abusive supervision behavior, is supported (β = 0.31, *p* < 0.001, M6). Third, as illustrated in Table 3, challenge stressors are significantly and positively correlated with ego depletion (β = 0.15, *p* < 0.05, M1), and hindrance stressors are also significantly and positively correlated with ego depletion (β = 0.39, *p* < 0.001, M1). In addition, ego depletion is significantly and positively correlated with abusive supervision behavior (β = 0.31, *p* < 0.001, M6). When ego depletion as the mediator variable is added into the model, ego depletion is significantly and positively correlated with abusive supervision behavior (β = 0.21, *p* < 0.001, M7). But the effects of challenge stressors on abusive supervision behavior (β = 0.09, *p* < 0.05, M7) and the effects of hindrance stressors on abusive supervision behavior (β = 0.14, *p* < 0.01, M7) are weakened.

**TABLE 3:** Results of hierarchical regression analyses.

Variables	ED				AS					
	M1	M2	M3	M4	M5	M6	M7	M8	M9	M10
	B	B	B	B	B	B	B	B	B	B
**Predictors**										
CS	0.15*	0.14*	0.18**	0.14*	0.12**		0.09*	0.09*	0.06	0.08
HS	0.39***	0.33***	0.31***	0.35***	0.22***		0.14**	0.12**	0.13**	0.10*
ED						0.31***	0.21***	0.18***	0.20***	0.21***
*EI*		−0.35***	−0.38***	−0.32***				−0.17*	–0.13	−0.18**
*CS* × *EI*			−0.25*						0.18*	
*HS* × *EI*				−0.26**						0.16*
Constant	1.37***	2.75***	2.78***	2.43***	1.50***	1.70***	1.21***	1.92***	1.85***	2.06***
**Controls**										
Gender	–0.03	–0.01	–0.02	–0.02	–0.10	–0.10	–0.09	–0.08	–0.08	–0.08
Age	0.00	0.00	0.00	0.00	0.00	0.00	0.00	0.00	0.00	0.002
Education	0.03	0.11	0.13	0.15	–0.03	–0.05	–0.04	0.00	–0.02	–0.03
Tenure	–0.01	0.00	0.01	0.01	0.01	0.01	0.01	0.01	0.01	0.01
*F*	12.19***	13.10***	12.43***	12.80***	8.88***	11.31***	11.10***	10.70***	10.32***	10.33***
*R*^2^	0.25	0.30	0.31	0.32	0.19	0.20	0.26	0.28	0.30	0.30
Δ*R*^2^	0.24***	0.05***	0.02*	0.03**	0.18***	0.19***	0.07***	0.02*	0.02*	0.02*

N = 228. Unstandardized regression coefficients are reported. Tenure = subordinate’s tenure with the supervisor. *p < 0.05, **p < 0.01, ***p < 0.001.

To further verify this mediating effect, we used Model 4 of the PROCESS macro with 5,000 resamples to test the indirect effect of challenge stressors and hindrance stressors on abusive supervision behavior via ego depletion. Results suggested that the indirect effect of challenge stressors on abusive supervision behavior through ego depletion (*b* = 0.03, boot SE = 0.02, 95% CI = [0.01, 0.07], excludes zero) is significant, and the indirect effect of hindrance stressors on abusive supervision behavior through ego depletion (*b* = 0.08, boot SE = 0.03, 95% CI = [0.03, 0.14], excludes zero) is also significant. These findings together provided statistical evidence for a mediating effect of ego depletion. Overall, Hypotheses 3a and 3b are supported.

**FIGURE 2:**
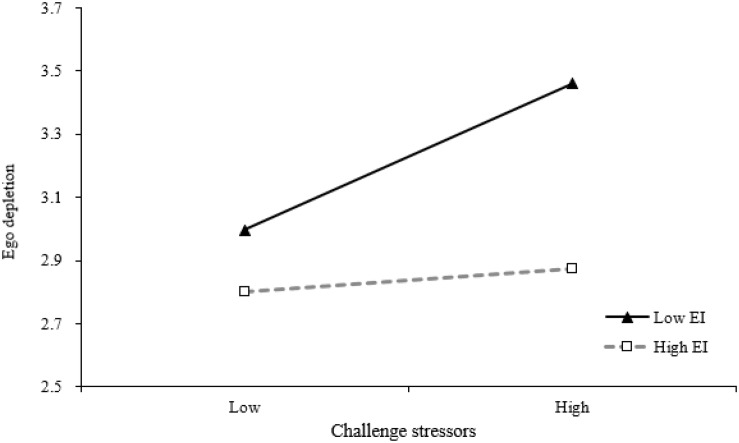
Interactive effects of challenge stressors and emotional intelligence (EI) on ego depletion.

**FIGURE 3:**
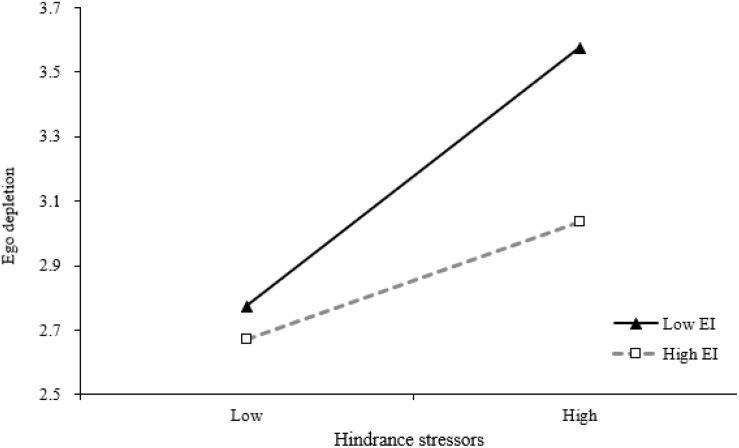
Interactive effects of hindrance stressors and EI on ego depletion.

Hypotheses 4a and 4b posited that EI would moderate the relationship between two different types of stressors (i.e., challenge stressors and hindrance stressors) and ego depletion, such that the relationship would be weaker (stronger) for supervisors high (low) in EI. Table 3 shows the results of the moderate role of EI. As shown in M3, the interaction of challenge stressors and EI is negatively and significantly related to ego depletion (β = −0.25, *p* < 0.05, M3). In order to clearly interpret the moderating effects, interaction effects are plotted. As shown in Figure 2, the positive relationship between challenge stressors and ego depletion is much more distinct in low EI (−1SD) rather than in high EI (+1SD). And results also suggest that the interaction of hindrance stressors and EI is negatively and significantly related to ego depletion (β = −0.26, *p* < 0.01, M4). In order to clearly interpret the moderating effects, interaction effects are plotted. As shown in Figure 3, the positive relationship between hindrance stressors and ego depletion is much more distinct in low EI (−1SD) than in high EI (+1SD). Therefore, Hypotheses 4a and 4b are supported.

Finally, we used Model 7 of the PROCESS macro with 5,000 resamples to generate bootstrap confidence intervals for the conditional indirect effect of two distinct categorized stressors (i.e., challenge stressors and hindrance stressors) on abusive supervision behavior via ego depletion at different levels of supervisors’ EI (see Table 4). For supervisors who had a high level of EI (+1SD), challenge stressors did not have a significant indirect effect on abusive supervision behavior through ego depletion (*b* = 0.010, boot SE = 0.014, 95%CI = [−0.017, 0.040], contains zero). For supervisors who had a low level of EI (−1SD), challenge stressors had a significant indirect effect on abusive supervision behavior through ego depletion (*b* = 0.065, boot SE = 0.036, 95%CI = [0.014, 0.154], excludes zero). And the pairwise contrasts between these conditional indirect effects were significant (*b* = −0.054, boot SE = 0.038, 95%CI = [−0.147, −0.001], excludes zero). Consequently, Hypothesis 5a is supported. Similarly, for supervisors who had a high level of EI (+1SD), hindrance stressors had a significant indirect effect on abusive supervision behavior through ego depletion (*b* = 0.046, boot SE = 0.020, 95%CI = [0.013, 0.092], excludes zero). For supervisors who had a low level of EI (−1SD), hindrance stressors also had a significant indirect effect on abusive supervision behavior through ego depletion (*b* = 0.101, boot SE = 0.034, 95%CI = [0.041, 0.174], excludes zero). And the pairwise contrasts between these conditional indirect effects were significant (*b* = −0.055, boot SE = 0.026, 95%CI = [−0.112, −0.012], excludes zero). Consequently, Hypothesis 5b is also supported.

**TABLE 4:** Indirect effect estimates with 95% confidence intervals.

Predictors	Estimate	Boot SE	LLCI	ULCI
**CS → ED → AS**				
Low EI (Effect1)	0.065	0.036	0.014	0.154
High EI (Effect2)	0.010	0.014	–0.017	0.040
Pairwise contrasts (Effect1–Effect2)	–0.054	0.038	–0.147	–0.001
**HS → ED → AS**				
Low EI (Effect1)	0.101	0.034	0.041	0.174
High EI (Effect2)	0.046	0.020	0.013	0.092
Pairwise contrasts (Effect1–Effect2)	–0.055	0.026	–0.112	–0.012

*N = 228. LLCL = lower limit of confidence interval; ULCL = upper limit of confidence interval.*

## Study Discussion

In our study, we developed and tested a model based on COR theory that attempted to probe why and when challenge and hindrance stressors have similar effects on abusive supervision behavior. As predicted, our findings first suggest that both challenge stressors and hindrance stressors have a positive effect on ego depletion, and ego depletion has a positive effect on abusive supervision behavior. Moreover, the findings also reveal that work stressors provoke ego depletion, which in turn triggers abusive supervision behavior. In this vein, the similar effects of challenge–hindrance stressors on abusive supervision behavior are instigated through ego depletion. Because ego depletion can well represent a state of self-regulatory resource depletion (Baumeister et al., 2007), as a result, our study reveal that self-regulatory resource depletion may be a key mediating mechanism that explains why two distinct categorized stressors have similar effects on abusive behavior (Barnes et al., 2015; Yam et al., 2016; Welsh et al., 2018). In addition, on the basis of COR, the present study also examined whether supervisors’ EI moderated the relationship between work stressors and ego depletion. Specifically, we found that the effect of challenge and hindrance stressors on abusive supervision behavior was weakened when the supervisor had a high level of EI. Finally, supervisors’ EI played a first-stage moderated-mediation role in the indirect effects of challenge stressors on abusive supervision and hindrance stressors on abusive supervision. That is, when the level of EI was high, the indirect effect of challenge stressors on abusive supervision behavior through ego depletion was weaker, and the indirect effect of hindrance stressors on abusive supervision behavior through ego depletion was also weaker. When the level of EI was low, the indirect effect of challenge stressors on abusive supervision behavior through ego depletion was stronger, and the indirect effect of hindrance stressors on abusive supervision behavior through ego depletion was also stronger. Because EI can well reflect individual differences in self-regulatory resources, as a result, our study reveal that resource replenishment may be a key mediating mechanism that explains when challenge–hindrance stressors have similar effects on abusive behavior.

## Conclusion

Drawing on COR theory, the current study developed and tested a model that explicates why and when challenge stressors and hindrance stressors lead to abusive supervision behavior. First, we found that both challenge stressors and hindrance stressors have a positive relationship to ego depletion, and ego depletion has a positive relationship to abusive supervision behavior. Second, we argued that resource depletion is the underlying mechanism of work stressors and abusive supervision behavior and testified to the mediating role of resource depletion between work stressors and abusive supervision behavior through a specific variable of psychological resource depletion: ego depletion. Finally, our findings suggested that EI, which is a characteristic variable and reflects individual differences in self-regulatory resources, is a moderating mechanism between challenge–hindrance stressors and ego depletion and has a first-stage moderated-mediation role in the indirect effects of these two distinct categorized stressors on abusive supervision. This study extends the current literature by directly testing self-regulatory resource depletion as a mediating mechanism and resource replenishment as a boundary condition of the effect of work stressors.

### Theoretical Implications

The current study provides several contributions to the existing literature. First, this study contributes to the enrichment of the relationship between challenge–hindrance stressors and abusive supervision. Although previous studies have found that work stressors are positively related to abusive supervision behavior (Burton et al., 2012; Mawritz et al., 2014; Eissa and Lester, 2017), knowledge about the similar effect of challenge–hindrance stressors on abusive supervision is limited. Furthermore, following the challenge–hindrance stressors framework, previous stress studies focused more on the different effects on some behaviors or attitudes and the similar effect on some psychology variables, ignoring that different categories of stressors may have a similar effect on the same behavior through similar psychological process. Although challenge stressors may result in some positive outcomes (i.e., enthusiasm, Wood and Michaelides, 2016; job performance, Wallace et al., 2009), this category is the same as hindrance stressors and will also consume individuals’ psychological resources when coped with. So, we reason that these two distinct categorized stressors may have a similar effect on abusive supervision behavior under the mechanism of self-regulatory resource depletion. Therefore, both challenge and hindrance stressors will consume the limited self-regulatory resources, resulting in the decline of self-regulation and in turn promoting the display of abusive supervision behavior. The current study expands the research on the similar effect of challenge–hindrance stressors on abusive supervision behavior and explains the intrinsic mechanism of the similar effect. Additionally, our findings also offer empirical evidentiary support for the self-regulation impairment explanation for abusive supervision (Tepper et al., 2017) and confirm that positive stressors (i.e., challenge stressors) may also result in negative leadership behavior (i.e., abusive supervision).

Second, we contribute to empirical research verifying the explanatory power of the resource depletion mechanism by focusing on a specific resource depletion variable: ego depletion. While previous studies have described the relationship between work stressors and abusive supervision behavior through negative emotions (Burton et al., 2012; Mawritz et al., 2014; Eissa and Lester, 2017), given that challenge stressors have been shown to evoke positive affect and negative affect (i.e., attentiveness and anxiety), while hindrance stressors have been shown to evoke negative affect (i.e., anger) (Rodell and Judge, 2009), the similar effect of two distinct categorized stressors on abusive supervision behavior cannot be discussed from the lens of emotional reaction. Our study predicts the relationship between challenge and hindrance stressors and abusive supervision behavior and supports the argument that the similar effects of work stressors are more likely to occur under resource depletion rather than emotional reaction. In our study, we found that ego depletion, a specific resource depletion variable, served as an important mediator linking work stressors to abusive supervision behavior. This suggests that both challenge stressors and hindrance stressors could drain supervisors’ self-regulatory resources at work and make them more likely to engage in abusive supervision behavior. Thus, our results show that resource depletion may be a key mechanism that explains why two distinct categorized stressors have similar effects on abusive behavior (Barnes et al., 2015; Yam et al., 2016; Welsh et al., 2018). Furthermore, our findings can offer empirical evidence that ego depletion can be viewed as a specific resource depletion variable.

Third, we enrich the existing research on EI by considering the resource replenishment mechanism as the boundary condition of the indirect relationship between work stressors and abusive supervision behavior. Although previous findings suggest that work stressors induce abusive supervision behavior, our findings show that this is not always the case from the lens of the resource. EI, first, itself is a kind of self-regulatory resource and, second, can help individuals gain more resources from playing roles successfully and interpersonal interaction. That is, EI can well reflect individual differences in self-regulatory resources. Therefore, EI enables individuals to effectively replenish the depletion of their self-regulatory resources. By examining the moderating effects of EI on the relationship between work stressors and ego depletion, our research reveals that EI plays a first-stage moderated-mediation role in the indirect effects of challenge stressors on abusive supervision and hindrance stressors on abusive supervision. Highlighting the role of EI, therefore, enriches the existing research by considering resource replenishment as the boundary condition of the indirect relationship between work stressors and abusive supervision behavior. Thus, our results show that resource replenishment may be a key mechanism that explains when challenge–hindrance stressors have similar effects on abusive behavior. Furthermore, our findings not only confirm the resource replenishment role of EI in stressful conditions but also confirm the existence and effectiveness of the resource replenishment mechanism. The logic of replenishment mechanisms can be used in stressful conditions to manage stress more effectively.

Finally, the present study applies the perspective of COR theory to understanding challenge–hindrance stressors and its negative consequences (i.e., ego depletion and abusive supervision behavior). This perspective provides a theoretical nuance that is particularly suitable for understanding the state of self-regulatory resource depletion as a reaction to work stressor change and for accounting for subsequent aggressive behaviors (Barnes et al., 2015; Lin et al., 2016). In addition, this perspective also provides evidence for the condition that individual differences in self-regulatory resources will affect the indirect effect of challenge–hindrance stressors on abusive supervision behavior. Accordingly, our study is theoretically driven by COR theory, thus helping people to gain a greater understanding of the complex relationship between two distinct categorized stressors and abusive supervision behavior and to comprehensively understand why and when distinct categorized stressors have a similar effect on abusive supervision behavior.

### Practical Implications

Our research also provides some guidance for managerial practice. First, our research identifies challenge and hindrance stressors as possible reasons for provoking abusive supervision behavior. In this vein, organizational decision-makers are well advised to be cautious about stressors in the work environment. It is widely believed that challenge stressors are often positively correlated with work performance. However, our study shows that challenge stressors can also lead to negative behavior. Therefore, organizational decision-makers should set appropriate indicators (i.e., a suitable workload or an attainable goal) for supervisors to reduce the negative impact of challenge stressors. In contrast, organizational decision-makers should minimize hindrance stressors. They should cut through red tape to improve efficiency and help supervisors maintain or protect their psychological resources. They should also provide clear role descriptions and effective communication about work roles to help supervisors reduce role conflict and role ambiguity.

Second, from the lens of resource depletion, the present study sheds light upon the mediating mechanism of ego depletion. This finding suggests that ego depletion transmits the detrimental effects of challenge and hindrance stressors. Thus, organizational decision-makers should pay more attention to creating opportunities for replenishment and recuperation of self-regulatory resources. The current study indicates that the direct way of conserving and replenishing self-regulatory resources is to reduce stressors in the work environment or to create appropriate channels for releasing stress. And other ways might include supporting more resources (e.g., more organizational support) for replenishment, providing enough time for recuperation, and so on.

Finally, our findings also showed that supervisors’ EI moderated the positive relationship between challenge and hindrance stressors and resource depletion, such that this relationship was strengthened only when supervisors’ EI was low (vs. high). And supervisors’ EI also moderated the indirect effect of challenge and hindrance stressors and abusive supervision behavior. As we discussed above, individuals with high EI can effectively replenish the continuous depletion of their self-regulatory resources when they cope with work stressors. Therefore, another means for lessening abusive supervision behavior is developing more self-regulatory resources through EI training. Organizational decision-makers might regularly provide EI training programs to help individuals gain more self-regulatory resources. Ways of training supervisors’ EI might include actually helping them to cognize their self-emotion and others’ emotion and to know how to use and regulate emotion.

### Limitations and Future Directions

Our study also has several limitations that provide directions for future research. First, we focused on the similar effect of challenge–hindrance stressors on abusive supervision behavior via resource depletion. However, according to the Affective Events Theory (AET) (Weiss and Cropanzano, 1996), challenge stressors can be viewed as positive work events, which in turn evoke more positive affect, while hindrance stressors can be viewed as negative work events, which in turn evoke more negative affect. Therefore, these two distinct categorized stressors may have different effects on abusive supervision behavior via different affect reactions. Future research should focus on why and when challenge–hindrance stressors have different effects on abusive supervision behavior.

Second, we designed a time-lagged study to probe the relationship between two different types of stressors and abusive supervision behavior. However, it is possible that other factors may influence supervisors’ stressors, ego depletion, and abusive supervision behavior, which we cannot control in our design. Future research using an experimental design may control many other factors in organizational situations, thus examining more definitive causal inferences between the variables in our model.

Finally, consistent with the research of Lin et al. (2016), we measured all variables at three time points in a week to avoid the retrospective bias of the long interval. The first limitation is that both independent variables and mediating variables came from the same source (self-report by supervisor) at the same time point and thus may have CMB (Podsakoff et al., 2012). In addition, although abusive supervision behavior in our study was reported by subordinates, results might deviate if such behavior were reported by the supervisor or by peers. Future research can measure abusive supervision behavior from three sources—supervisor, subordinate, and peers—and discuss the difference among them.

## Data Availability Statement

The datasets generated for this study are available on request to the corresponding author.

## Ethics Statement

All study procedures were approved by the Ethics Committee of the Guangdong University of Technology, and informed consent of the participation was implied through survey completion.

## Author Contributions

ZL and BH conceived and designed the study. ZL collected and analyzed the data. ZL and XS interpreted the data and drafted the manuscript. BH, ZL, XS, and YZ reviewed and edited the manuscript. XS administered the project.

## Conflict of Interest

The authors declare that the research was conducted in the absence of any commercial or financial relationships that could be construed as a potential conflict of interest.
