# Supplementary material for: Demonstrating the Psychological Aspects of Stressors and Abusive Supervision Behavior: Attainment of Sustainability Under the Rubric of Resources Theory
Source: Front Psychol. 2020 Mar 13;11:293. doi: 10.3389/fpsyg.2020.00293 (PMC7083137; doi:10.3389/fpsyg.2020.00293)
Supplement: Supplementary file 1 [file Data_Sheet_1.docx]

# Appendix

Measurements of research model constructs

| Survey 1 (T1) | |  | |  |
| --- | --- | --- | --- | --- |
| Please indicate to what extent you agree with the following statements (1 = strongly disagree to 5 = strongly agree). | | Loading | | Reference |
| Emotional intelligence  (Cronbach’s  = 0.93; KMO =0.92; AVE =0.4709; CR=0.9341) | | | | |
| Self-emotion appraisal (SEA) | I have a good sense of why I have certain feelings most of the time. | 0.66 | 0.73 | Wong and Law, 2002;  Mayer et al., 2008; Law et al., 2004 |
|  | I have good understanding of my own emotions. | 0.71 |  |  |
|  | I really understand what I feel. | 0.67 |  |  |
|  | I always know whether or not I am happy. | 0.71 |  |  |
| Others’ emotion appraisal (OEA) | I always know my friends' emotions from their behavior. | 0.62 | 0.78 |  |
|  | I am a good observer of others' emotions. | 0.69 |  |  |
|  | I am sensitive to the feelings and emotions of others. | 0.75 |  |  |
|  | I have good understanding of the emotions of people around me. | 0.75 |  |  |
| Use of emotion (UOE) | I always set goals for myself and then try my best to achieve them. | 0.73 | 0.79 |  |
|  | I always tell myself I am a competent person. | 0.73 |  |  |
|  | I am a self-motivated person. | 0.69 |  |  |
|  | I would always encourage myself to try my best. | 0.70 |  |  |
| Regulation of emotion (ROE) | I am able to control my temper and handle difficulties rationally. | 0.71 | 0.69 |  |
|  | I am quite capable of controlling my own emotions. | 0.60 |  |  |
|  | I can always calm down quickly when I am very angry. | 0.57 |  |  |
|  | I have good control of my own emotions. | 0.66 |  |  |
| Survey 2 (T2) | |  | |  |
| Please indicate to what extent you agree with the following statements about produced stress at work during the past 2 days (1=no stress to 5=a great deal of stress). | | Loading | | Reference |
| Challenge stressors  (Cronbach’s  =0.88; KMO =0.87; AVE =0.5583; CR=0.8829) | | | | |
| The number of projects and or assignments I have. | | 0.83 | | Cavanaugh et al., 2000;  Wallace et al., 2004 |
| The amount of time I spend at work. | | 0.78 | |  |
| The volume of work that must be accomplished in the allotted time. | | 0.78 | |  |
| Time pressure I experience. | | 0.72 | |  |
| The amount of responsibility I have. | | 0.70 | |  |
| The scope of responsibility my position entails. | | 0.66 | |  |
| Hindrance stressors  (Cronbach’s  =0.86; KMO = 0.86; AVE =0.5436; CR=0.8558) | | | |  |
| The degree to which politics rather than performance affects organizational decisions. | | 0.76 | |  |
| The inability to clearly understand what is expected of me on the job. | | 0.79 | |  |
| The amount of red tape I need to get through to get my job done. | | 0.67 | |  |
| The lack of job security I have. | | 0.76 | |  |
| The degree to which my career seems “stalled.” | | 0.70 | |  |
| Please indicate to what extent you agree with the following statements about your state of psychological resource or your psychological feeling at work during the past 2 days (1 = very slightly or not at all to 5 = very much). | | Loading | | Reference |
| Ego depletion  (Cronbach’s  =0.89; KMO = 0.87; AVE =0.6154; CR=0.8888) | | | |  |
| I feel drained. | | 0.77 | | Lin and Johnson, 2015;  Lin et al., 2016;  Johnson et al., 2014; Lanaj et al., 2014 |
| My mind feels unfocused. | | 0.83 | |  |
| It would take a lot of effort for me to concentrate on something. | | 0.77 | |  |
| My mental energy is running low. | | 0.80 | |  |
| I feel like my willpower is gone. | | 0.75 | |  |
| Survey 3 (T3) | |  | |  |
| Please indicate how often your supervisor engaged in each behavior during the past 3 days (1 = *never* to 5 =*very often*). | | Loading | | Reference |
| Abusive supervision behavior  (Cronbach’s  =0.81; KMO =0.82; AVE =0.4625; CR=0.8086) | | | |  |
| My supervisor ridicules me. | | 0.75 | | Mitchell and Ambrose, 2007 |
| My supervisor tells me my thoughts or feelings are stupid. | | 0.60 | |  |
| My supervisor puts me down in front of others. | | 0.78 | |  |
| My supervisor makes negative comments about me to others. | | 0.70 | |  |
| My supervisor tells me I'm incompetent. | | 0.54 | |  |
